# Association of Multimorbidity and Excess Mortality After Fractures Among Danish Adults

**DOI:** 10.1001/jamanetworkopen.2022.35856

**Published:** 2022-10-10

**Authors:** Thach Tran, Dana Bliuc, Thao Ho-Le, Bo Abrahamsen, Joop P. van den Bergh, Weiwen Chen, John A. Eisman, Piet Geusens, Louise Hansen, Peter Vestergaard, Tuan V. Nguyen, Robert D. Blank, Jacqueline R. Center

**Affiliations:** 1 Skeletal Diseases Program, Garvan Institute of Medical Research, Sydney, New South Wales, Australia; 2 Faculty of Medicine, University of New South Wales, Sydney, New South Wales, Australia; 3 School of Biomedical Engineering, University of Technology, Sydney, New South Wales, Australia; 4 Faculty of Engineering and Information Technology, Ha Tinh University, Ha Tinh, Vietnam; 5 Department of Medicine, Holbæk Hospital, Holbæk, Denmark; 6 Department of Clinical Research, Odense Patient Data Explorative Network, University of Southern Denmark, Odense, Denmark; 7 Research School NUTRIM (Nutrition and Translational Research in Metabolism), Subdivision of Rheumatology, Department of Internal Medicine, Maastricht University Medical Center, Maastricht, the Netherlands; 8 Department of Internal Medicine, VieCuri Medical Center of Noord-Limburg, Venlo, the Netherlands; 9 School of Medicine Sydney, University of Notre Dame Australia, Sydney, New South Wales; 10 Research School CAPHRI (Care and Public Health Research Institute), Subdivision of Rheumatology, Department of Internal Medicine, Maastricht University Medical Center, Maastricht, the Netherlands; 11 Biomedical Research Institute, University Hasselt, Hasselt, Belgium; 12 Kontraktenheden, North Denmark Region, Aalborg, Denmark; 13 Department of Clinical Medicine, Aalborg University, Aalborg, Denmark; 14 Department of Endocrinology, Aalborg University Hospital, Aalborg, Denmark; 15 Steno Diabetes Center North Jutland, Aalborg, Denmark

## Abstract

**Importance:**

Limited knowledge about interactions among health disorders impedes optimal patient care. Because comorbidities are common among patients 50 years and older with fractures, these fractures provide a useful setting for studying interactions among disorders.

**Objective:**

To define multimorbidity clusters at the time of fracture and quantify the interaction between multimorbidity and fracture in association with postfracture excess mortality.

**Design, Setting, and Participants:**

This nationwide cohort study included 307 870 adults in Denmark born on or before January 1, 1951, who had an incident low-trauma fracture between January 1, 2001, and December 31, 2014, and were followed up through December 31, 2016. Data were analyzed from February 1 to March 31, 2022.

**Main Outcomes and Measures:**

Fracture and 32 predefined chronic diseases recorded within 5 years before the index fracture were identified from the Danish National Hospital Discharge Register. Death was ascertained from the Danish Register on Causes of Death. Latent class analysis was conducted to identify multimorbidity clusters. Relative survival analysis was used to quantify excess mortality associated with the combination of multimorbidity and fractures at specific sites.

**Results:**

Among the 307 870 participants identified with incident fractures, 95 372 were men (31.0%; mean [SD] age at fracture, 72.3 [11.2] years) and 212 498 were women (69.0%; mean [SD] age at fracture, 74.9 [11.2] years). During a median of 6.5 (IQR, 3.0-11.0) years of follow-up, 41 017 men (43.0%) and 81 727 women (38.5%) died. Almost half of patients with fractures (42.9%) had at least 2 comorbidities. Comorbidities at fracture were categorized as low-multimorbidity (60.5% in men and 66.5% in women), cardiovascular (23.7% in men and 23.5% in women), diabetic (5.6% in men and 5.0% in women), malignant (5.1% in men and 5.0% in women), and mixed hepatic and/or inflammatory (5.1% in men only) clusters. These clusters distinguished individuals with advanced, complex, or late-stage disease from those with earlier-stage disease. Multimorbidity and proximal or lower leg fractures were associated with increased mortality risk, with the highest excess mortality found in patients with hip fracture in the malignant cluster (1-year excess mortality: 40.8% [95% CI: 38.1%-43.6%]). The combination of multimorbidity and fracture compounded the association with mortality, conferring much greater risk than either alone.

**Conclusions and Relevance:**

Concomitant illnesses were common and clustered into distinct multimorbidity clusters that were associated with excess postfracture mortality. The compound contribution of multimorbidity to postfracture excess mortality highlights the need for more comprehensive approaches in these high-risk patients. The analytical approach applied to fracture could also be used to examine other sentinel health events.

## Key Points

**Question:**

Are there associations between multimorbidity and excess mortality among older adults with fractures?

**Findings:**

This nationwide cohort study of 307 870 adults 50 years or older with an incident fracture found chronic health disorders were common and grouped into distinct multimorbidity clusters, providing additional stratification beyond simple additive summation of diagnoses. The combination of specific multimorbidity clusters and proximal fractures was associated with a compounding mortality, conferring much greater risk of mortality than either event alone.

**Meaning:**

The compound contribution of multimorbidity to postfracture mortality highlights the need for more comprehensive approaches in patients with multimorbidity; the analytical approach used for fracture can be applied to other sentinel health events.

## Teaser

This cohort study identifies clusters of multimorbidity occurring among patients with fractures and quantifies the association of multimorbidity clusters and specific fractures with mortality risk.

## Introduction

Multimorbidity is the simultaneous presence of 2 or more chronic health disorders.^1^ As the world population ages, multimorbidity is becoming more common; approximately 65% of patients 65 years or older and 80% of those 85 years or older have at least 2 chronic health conditions.^2^ Most current disease management guidelines have limited applicability to this high-risk group,^3,4,5^ because they are based on randomized clinical trials that typically exclude individuals with multimorbidity. Consequently, evidence on optimal care for people with multimorbidity is inadequate.^6^ Identifying distinct and reliable clusters of chronic health conditions that frequently co-occur is a logical step to address this need.^4,7^

Fractures provide an informative setting in which to study outcomes associated with multimorbidity. Half of patients with fractures have at least 2 comorbidities.^8^ The contribution of multimorbidity to mortality after hip fracture is well illustrated by chronic obstructive pulmonary disease.^9,10^ Patients with hip fracture and without chronic obstructive pulmonary disease had an associated 3-fold increased mortality risk, whereas those with chronic obstructive pulmonary disease experienced as much as a 6-fold increased risk compared with matched controls with neither condition.^10^ Less is known about the interaction between multimorbidity and nonhip fractures, which may have an even greater population association with mortality risk.^11^ Identifying distinct coexisting chronic disease clusters that are associated with postfracture mortality risk would facilitate delivery of individualized care.

We undertook a nationwide cohort study of fractures and comorbidities to (1) identify clusters of multimorbidity occurring in patients with fractures and (2) quantify the association of multimorbidity clusters and specific fractures in combination with mortality risk. Using fracture as an example, these analyses can inform the more general problem of multimorbidity.

## Methods

### Study Design

This population-based cohort study included all individuals in Denmark born on or before January 1, 1951, who sustained an initial incident fracture between January 1, 2001, and December 31, 2014. We excluded face, skull, and digit fractures and high-trauma fractures due to traffic accidents. Individuals with a low-trauma fracture at 45 years or older between January 1, 1996, and December 31, 1999, were also excluded to avoid potential bias arising from the incident fracture being a second fracture. The initial incident fracture was defined as the first low-trauma fracture reported during the study period. We used codes from the *International Statistical Classification of Disease and Related Health Problems, Tenth Revision*, to identify individuals with specific proximal fractures of the hip, femur, pelvis, vertebrae, humerus, rib, and clavicle; distal fractures of the forearm, lower leg, knee, ankle, and foot and hand; and comorbidities from the Danish National Hospital Discharge Register (NHDR) (eTable 1 in the Supplement). When multiple fractures occurred during a single event, only the most proximal fracture was considered. The predefined comorbidities included 32 chronic health disorders documented within 5 years before the index fracture, which have been shown to be robust in mortality research^12^ and appropriate for use with administrative data.^13,14^ The study was approved by the National Board of Health, the Danish Data Protection Agency, and Statistics Denmark, and subject to independent control and monitoring by the Danish Health Data Authority. Written informed consent was waived owing to the use of routinely collected, pseudonymized registry data. This study followed the Strengthening the Reporting of Observational Studies in Epidemiology (STROBE) reporting guideline.

Study participants were followed up for mortality to December 31, 2016, allowing at least 2 years of postfracture follow-up. Death was ascertained from the Danish Register on Causes of Death.

### Statistical Analysis

Sex-specific analyses were performed from February 1 to March 31, 2022. Latent class analysis was performed to identify distinct multimorbidity clusters present at the time of fracture, accounting for all possible combinations of specific comorbidities.^15^ Latent class analysis is a clustering technique that uses maximum likelihood estimation to assimilate the observed comorbidities into unobserved classes that are statistically distinct and clinically meaningful.^16^ Despite being agnostic to biological mechanisms, latent class analysis could group comorbidities into unique complex constructs that cannot be measured directly but share a plausible common pathophysiological mechanism, genetic propensity, or environmental exposure, statistically known as *latent classes*.^17^ The optimal latent class model provides the best fit for the data^18^ and the best separation among classes^19^ while simultaneously including at least 5% in the smallest class^18^ (eMethods in the Supplement).

Once the optimal latent class model was selected, each participant was assigned to the best-fit cluster for which he or she had the highest computed probability of membership. Characteristics at the time of fracture were described according to each multimorbidity cluster; the between-cluster differences were analyzed using χ^2^ and analysis of variance tests with Tukey correction for multiple comparisons where appropriate.

Relative survival analysis was conducted to quantify excess mortality attributable to the combination of specific multimorbidity clusters and individual fracture sites, accounting for the confounding effects of sex, aging, and time-related mortality changes in the background population.^20^ Excess mortality for patients in a specific multimorbidity cluster with fractures at a specific site, calculated as 1 minus its corresponding relative survival ratio, can be interpreted as the proportion of patients who would die for the combination between the specific comorbidity cluster and the individual fracture site. The relative survival ratio is the ratio of observed survival in a cohort of individuals in a specific multimorbidity cluster with fractures at a specific site to expected survival in a comparable general population.^20^ The observed survival was calculated using all-cause deaths in the fracture cohort of individuals with a specific fracture and in a specific multimorbidity cluster, whereas the expected survival was the survival probability of similar individuals, derived from the general population of the same age, sex, and calendar period as the fracture cohort^20,21^ using the Ederer II method (eMethods in the Supplement). Analyses were performed using Stata, version 16 (StataCorp LLC) and R, version 4.1.2, on a Window platform (R Foundation for Statistical Computing). A 2-sided *P* < .05 was considered statistically significant.

## Results

An initial incident fracture occurred in 307 870 patients, including 95 372 men (31.0%) at a mean (SD) age of 72.3 (11.2) years and 212 498 women (69.0%) at a mean age of 74.9 (11.2) years (eFigure 1 in the Supplement). Forearm, hip, and humerus fractures were the most common fractures, together contributing 53.5% and 69.8% of fractures in men and women, respectively (eTable 2 in the Supplement). Men had more comorbidities than women (median: 2.0 [IQR, 0-3.0)] vs 1.0 [IQR, 0-2.0]).

Comorbidity was highly prevalent at the time of fracture, with 42.9% of patients overall (50.7% of men and 39.4% of women) having 2 or more comorbidities, among which the most common conditions were cardiovascular diseases (77.2% in men and 72.8% in women), cancer (33.9% in men and 32.1% in women), hypertension (30.3% in men and 34.8% in women), and chronic lung disease (28.2% in men and 27.3% in women). Patients with proximal fractures had more comorbidities than those with distal fractures (eTable 2 in the Supplement).

### Distinct Clusters of Multimorbidity at the Time of Fracture

Latent class analysis revealed 5- and 4-multimorbidity class models as optimal for representing multimorbidity clusters in men and women, respectively (eTable 3 and eFigure 2 in the Supplement). These clusters, named according to the predominant diseases, were low multimorbidity (60.5% in men and 66.5% in women), cardiovascular (23.7% in men and 23.5% in women), diabetic (5.6% in men and 5.0% in women), malignant (5.1% in men and 5.0% in women), and a mixed cluster of hepatic and/or inflammatory (5.1% in men only) (Table 1). The probabilities that an individual in a specific multimorbidity cluster had specific health disorders are presented in eTable 4 in the Supplement.

**Table 1.** Participant Characteristics at Time of Fracture by Multimorbidity Clusters

Characteristics	Multimorbidity disease cluster^a^								
	Men (n = 95 372)					Women (n =212 498)			
	Low multimorbidity	Cardiovascular	Diabetic	Malignant	Hepatic and/or inflammatory^b^	Low multimorbidity	Cardiovascular	Diabetic	Malignant
All	57 711 (60.5**)**	22 589 (23.7)	5315 (5.6**)**	4907 (5.1)	4850 (5.1)	141 222 (66.5)	49 932 (23.5)	10 697 (5.0)	10 647 (5.0)
Age at fracture, mean (SD), y	70.2 (11.3)	78.2 (9.8)	72.0 (9.3)	72.8 (9.4)	70.6 (10.3)	73.1 (11.3)	80.2 (9.7)	75.5 (9.6)	73.2 (9.7)
Comorbidities, median (IQR) No.	1.0 (0-1.0)	4.0 (3.0-5.0)	5.0 (4.0-7.0)	3.0 (2.0-4.0)	4.0 (3.0-5.0)	0 (0-1.0)	3.0 (2.0-4.0)	4.0 (3.0-6.0)	3.0 (2.0-3.0)
No. of comorbidities									
0	26 632 (46.1)	0	0	0	0	73 740 (52.2)	0	0	0
1	20 156 (34.9)	0	0	0	272 (5.6)	53 124 (37.6)	390 (0.8)	0	0
2	9874 (17.1)	3612 (16.0)	302 (5.7)	1875 (38.2)	802 (16.5)	13 812 (9.8)	12 911 (25.9)	1650 (15.4)	4234 (39.8)
3-4	1049 (1.8)	12 569 (55.6)	1602 (30.1)	2412 (49.1)	2206 (45.5)	546 (0.4)	26 206 (52.5)	3946 (36.9)	5418 (50.9)
≥5	0	6408 (28.4)	3411 (64.2)	620 (12.6)	1570 (32.4)	0	10 425 (20.9)	5101 (47.7)	995 (9.3)
Specific comorbidities^c^									
Myocardial infarction	2139 (3.7)	6675 (29.5)	1434 (27.0)	335 (6.8)	568 (11.7)	1990 (1.4)	9322 (18.7)	2111 (19.7)	258 (2.4)
Congestive heart failure	714 (1.2)	9799 (43.4)	1872 (35.2)	498 (10.1)	887 (18.3)	2044 (1.4)	16 261 (32.6)	2968 (27.7)	423 (4.0)
Peripheral vascular disease	2071 (3.6)	4833 (21.4)	1947 (36.6)	445 (9.1)	604 (12.5)	3463 (2.5)	7247 (14.5)	2262 (21.1)	544 (5.1)
Cerebrovascular disease	7266 (12.6)	9862 (43.7)	2070 (38.9)	584 (11.9)	1252 (25.8)	10 250 (7.3)	19 633 (39.3)	3868 (36.1)	2665 (25.0)
Cardiac valvular disease	115 (0.2)	2097 (9.3)	309 (5.8)	110 (2.2)	181 (3.7)	296 (0.2)	4456 (8.9)	520 (4.9)	155 (1.5)
Cardiac arrhythmias	1302 (2.3)	8854 (39.2)	1184 (22.3)	357 (7.3)	748 (15.4)	2388 (1.7)	14 793 (29.6)	1766 (16.5)	460 (4.3)
Diabetes without chronic complications	2632 (4.6)	2907 (12.9)	5146 (96.8)	621 (12.7)	779 (16.1)	3737 (2.6)	3343 (6.7)	10 293 (96.2)	1171 (11.0)
Diabetes with chronic complications	210 (0.4)	66 (0.3)	4744 (89.3)	12 (0.2)	135 (2.8)	227 (0.2)	0	5862 (54.8)	6 (0.1)
Any malignant neoplasm except skin	8210 (14.2)	4997 (22.1)	1156 (21.7)	4907 (100)	1109 (22.9)	16 896 (12.0)	7857 (15.7)	3008 (28.1)	10 647 (100)
Metastatic solid tumor	188 (0.3)	175 (0.8)	186 (3.5)	3324 (67.7)	167 (3.4)	386 (0.3)	309 (0.6)	222 (2.1)	5936 (55.7)
Rheumatic or rheumatoid arthritis or collagen vascular diseases	445 (0.8)	186 (0.8)	265 (5.0)	39 (0.8)	2390 (49.3)	5983 (4.2)	4931 (9.9)	1039 (9.7)	794 (7.5)
Mild liver disease	484 (0.8)	93 (0.4)	278 (5.2)	88 (1.8)	1976 (40.7)	982 (0.7)	1706 (3.4)	552 (5.2)	356 (3.3)
Moderate or severe liver disease	32 (0.1)	8 (0.03)	100 (1.9)	16 (0.3)	1052 (21.7)	53 (0.03)	673 (1.3)	203 (1.9)	136 (1.3)
Hypertension	2250 (3.9)	8779 (38.9)	2526 (47.5)	1078 (22.0)	963 (19.9)	6167 (4.4)	21 136 (42.3)	4734 (44.3)	1679 (15.8)
Chronic pulmonary disease	4789 (8.3)	7394 (32.7)	1233 (23.2)	864 (17.6)	1191 (24.5)	10 331 (7.3)	14 276 (28.6)	2328 (21.8)	2168 (20.4)
Pulmonary circulation disorders	138 (0.2)	671 (3.0)	109 (2.1)	106 (2.2)	69 (1.4)	158 (0.1)	1542 (3.1)	184 (1.7)	169 (1.6)
Dementia	3487 (6.0)	4168 (18.5)	575 (10.8)	102 (2.1)	439 (9.1)	8326 (5.9)	10 455 (20.9)	1146 (10.7)	388 (3.6)
Psychoses	164 (0.3)	163 (0.7)	26 (0.5)	13 (0.3)	35 (0.7)	203 (0.1)	439 (0.9)	56 (0.5)	29 (0.3)
Depression	283 (0.5)	1487 (6.6)	219 (4.1)	87 (1.8)	278 (5.7)	397 (0.3)	5067 (10.1)	420 (3.9)	312 (2.9)
Paralysis or hemiparaplegia	344 (0.6)	340 (1.5)	94 (1.8)	97 (2.0)	66 (1.4)	402 (0.3)	423 (0.8)	86 (0.8)	141 (1.3)
Neurological disorders	1881 (3.3)	2108 (9.3)	265 (5.0)	105 (2.1)	344 (7.1)	2372 (1.7)	3227 (6.5)	357 (3.3)	302 (2.8)
Peptic ulcer disease	2197 (3.8)	3575 (15.8)	733 (13.8)	475 (9.7)	1064 (21.9)	4307 (3.0)	7839 (15.7)	1284 (12.0)	816 (7.7)
Chronic renal disease	357 (0.6)	3277 (14.5)	1362 (25.6)	377 (7.7)	386 (8.0)	428 (0.3)	3634 (7.3)	1749 (16.3)	451 (4.2)
Hypothyroidism	82 (0.1)	325 (1.4)	83 (1.6)	40 (0.8)	46 (0.9)	794 (0.6)	2645 (5.3)	625 (5.8)	230 (2.2)
Coagulopathy	46 (0.1)	257 (1.1)	51 (1.0)	62 (1.3)	112 (2.3)	108 (0.1)	407 (0.8)	96 (0.9)	70 (0.7)
Obesity	135 (0.2)	425 (1.9)	594 (11.2)	62 (1.3)	103 (2.1)	447 (0.3)	1040 (2.1)	1223 (11.4)	319 (3.0)
Unintended weight loss	60 (0.1)	321 (1.4)	70 (1.3)	75 (1.5)	96 (2.0)	89 (0.1)	923 (1.8)	90 (0.8)	89 (0.8)
Fluid or electrolyte disorders	634 (1.1)	3055 (13.5)	507 (9.5)	208 (4.2)	443 (9.1)	937 (0.7)	8773 (17.6)	890 (8.3)	556 (5.2)
Anemia	118 (0.2)	974 (4.3)	175 (3.3)	106 (2.2)	213 (4.4)	285 (0.2)	2562 (5.1)	301 (2.8)	200 (1.9)
Alcohol abuse	215 (0.4)	72 (0.3)	117 (2.2)	24 (0.5)	1409 (29.1)	72 (0.1)	794 (1.6)	147 (1.4)	86 (0.8)
Drug abuse	45 (0.1)	71 (0.3)	18 (0.3)	4 (0.1)	58 (1.2)	29 (0.02)	273 (0.5)	18 (0.2)	10 (0.1)
HIV/AIDS	27 (0.05)	17 (0.1)	3 (0.1)	5 (0.1)	8 (0.2)	7 (0.01)	2 (0.004)	2 (0.02)	5 (0.05)

^a^ Data are presented as No. (%), unless indicated otherwise.

^b^ Data were collected for men only.

^c^ Includes not only chronic diseases that require hospitalization, but also those documented as either secondary diagnoses or at outpatient or emergency visits.

The low-multimorbidity cluster included 60.5% of men and 66.5% of women with fractures. Men in this cluster sustained fracture at a mean (SD) age of 70.2 (11.3) years and women sustained fracture at a mean (SD) age of 73.1 (11.3) years (Table 1). Among individuals in this cluster, 46.1% of men and 52.2% of women had no comorbidity, and 34.9% of men and 37.6% of women had only 1 comorbidity. The most common comorbidities included cancer (14.2% in men and 12.0% in women) and stroke (12.6% in men and 7.3% in women).

Almost one-quarter of patients with fractures were grouped in the cardiovascular cluster (23.7% in men and 23.5% in women), in which 84.0% of men and 73.4% of women had 3 or more comorbidities. Unsurprisingly, almost all patients in this group had at least 1 cardiovascular condition, with the most common being stroke (43.7% in men and 39.3% in women), congestive heart failure (43.4% in men and 32.6% in women), and myocardial infarction (29.5% in men and 18.7% in women). Compared with individuals with cardiovascular diseases in the low-multimorbidity cluster (6.7% in men and 4.5% in women), those in the cardiovascular cluster were older (mean [SD] age, 78.2 [9.8] vs 73.5 [10.8] years in men; 80.2 [9.7] vs 77.4 [10.6] in women) and had more comorbidities (median: 4.0 [IQR, 3.0-5.0] in men and 3.0 [IQR, 2.0-4.0] in women vs 1.0 [IQR, 1.0-2.0] in those with cardiovascular diseases in the low-multimorbidity cluster).

In addition to these 2 major multimorbidity clusters, patients with fractures were also grouped into other smaller clusters. All patients in the diabetic and malignant clusters had at least 1 diagnosis of diabetes and cancer at the time of fracture, respectively. They were older and had more comorbidities than individuals in the low-multimorbidity cluster who also had diabetes or cancer, respectively. Notably, patients with fractures in the diabetic cluster had the highest number of comorbidities (median: 5.0 [IQR, 4.0-7.0] in men and 4.0 [IQR, 3.0-6.0] in women), with 64.2% of men and 47.7% of women having 5 or more comorbidities. Three-quarters of patients in the diabetic cluster and one-third of those in the malignant cluster had cardiovascular diseases. The diabetic and malignant clusters, although each constituting approximately 5% of the population, captured 94.2% of all cases of diabetes with chronic complications and 85.0% of all metastatic cancers among patients with fractures, respectively. Importantly, 80.6% of men with chronic renal diseases were included in the cardiovascular and diabetic clusters (ie, 56.9% in the cardiovascular cluster and 23.6% in the diabetic cluster). Similarly, 85.9% of women with renal diseases were included in 2 clusters (cardiovascular and diabetic clusters).

Unlike women, 5.1% of men were grouped into a hepatic and/or inflammatory cluster, whose most common conditions were rheumatoid arthritis or collagen vascular diseases (49.3%), liver diseases (46.0%), or alcohol abuse (29.1%). Although men in this cluster sustained fracture at an age similar to those in the low-multimorbidity class (mean [SD] age, 70.6 [10.3] vs 70.2 [11.3] years), they had significantly more comorbidities (median, 4.0 [IQR, 3.0-5.0] vs 1.0 [IQR, 0-1.0]). Notably, this cluster captured 71.8% of rheumatoid arthritis, 67.7% of mild and 87.1% of moderate to severe liver disease, and 76.7% of alcohol abuse cases reported among men with fractures.

**Figure 1.**
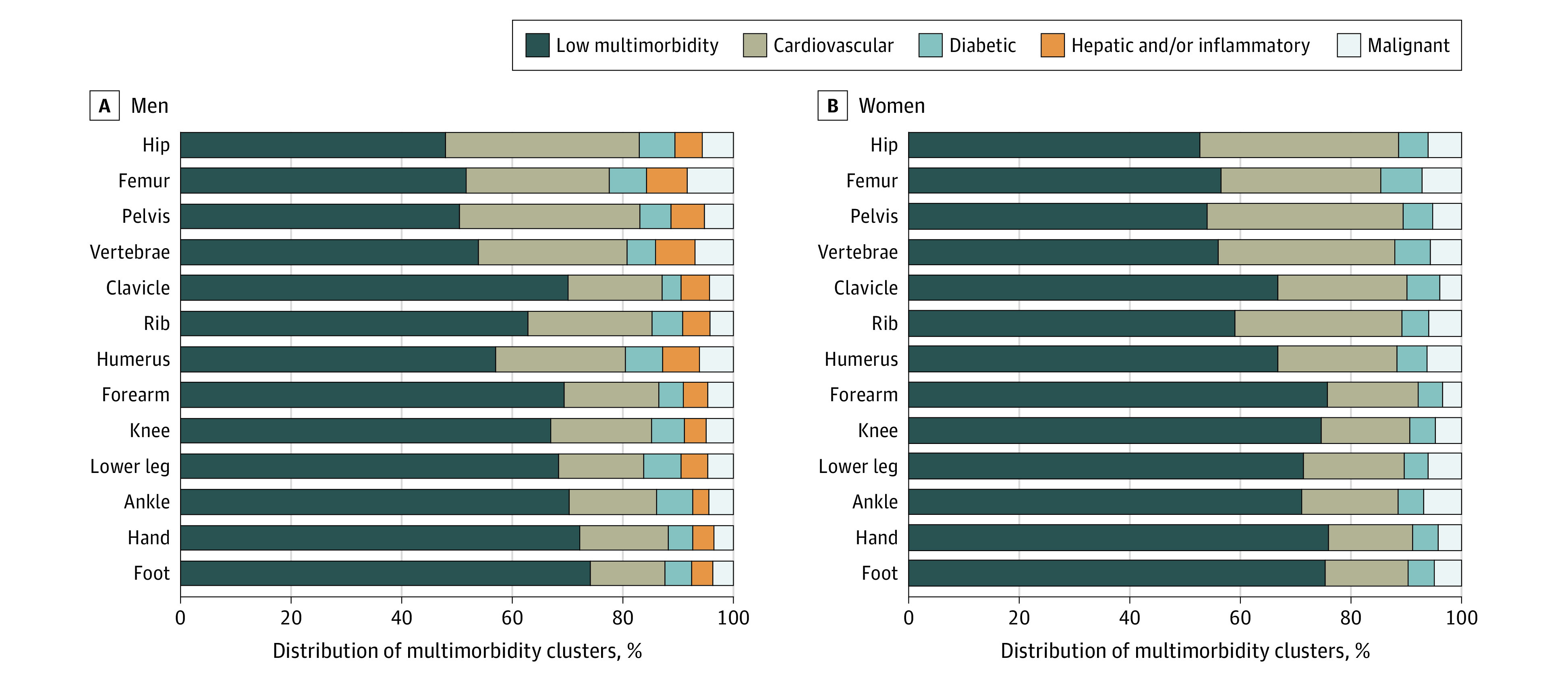
Distribution of Multimorbidity Clusters by Specific Fracture Sites

The distribution of fractures was multimorbidity cluster–specific (Figure 1). Patients with distal fractures were more likely to be in the low-multimorbidity cluster than those with hip or other proximal fractures. Almost 75% of hand or foot fractures occurred in the low-multimorbidity cluster, compared with half of hip fractures (47.9% in men and 52.6% in women). In contrast, one-third of patients with hip fracture were in the cardiovascular cluster, compared with 16.1% of distal fractures and 25.8% of nonhip proximal fractures.

### Association of the Interaction Between Multimorbidity Clusters and Fracture Sites With Postfracture Mortality

**Figure 2.**
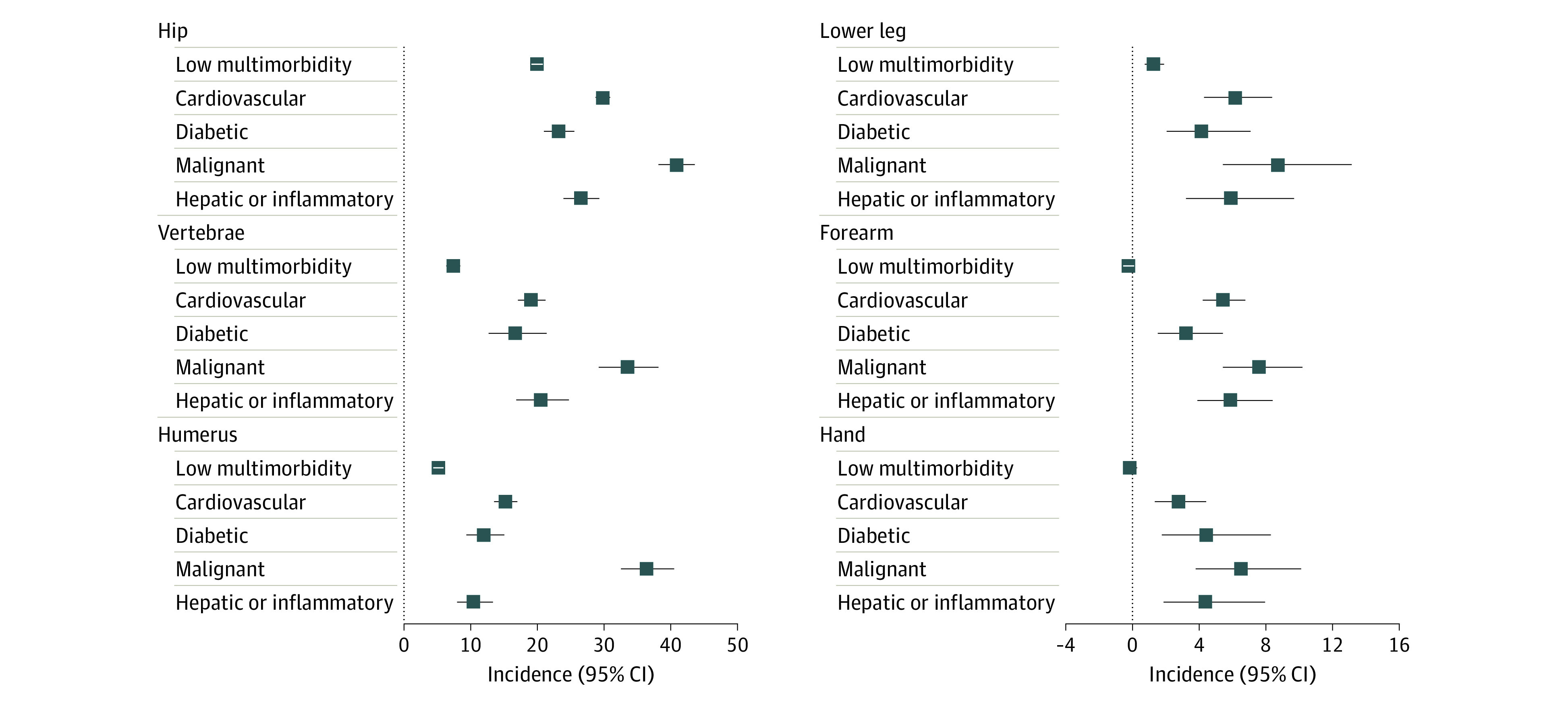
Excess Mortality 1 Year After Fracture in Selected Sites by Specific Multimorbidity Clusters Compared With the Age-, Sex-, and Calendar Period–Matched General Population Among Men Error bars indicate 95% CI.

**Figure 3.**
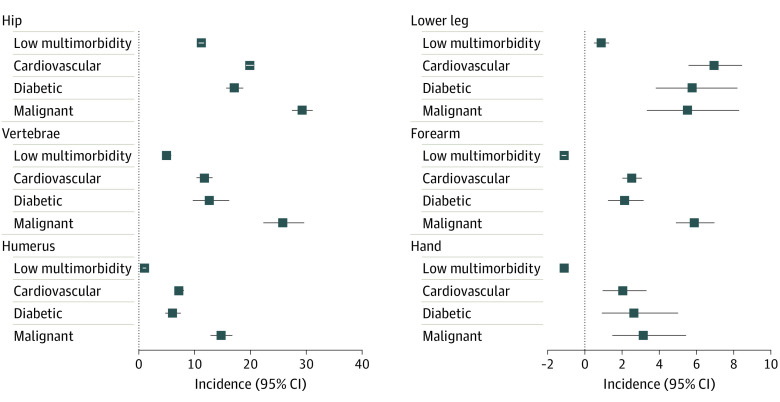
Excess Mortality 1 Year After Fracture in Selected Sites by Specific Multimorbidity Clusters Compared With the Age-, Sex-, and Calendar Period–Matched General Population Among Women Error bars indicate 95% CI.

During a median follow-up of 6.5 (IQR, 3.0-11.0) years (6.0 [IQR, 2.5-10.0] years in men and 6.7 [IQR, 3.3-10.8] years in women), 41 017 men (43.0%) and 81 727 women (38.5%) died, yielding mortality incidence rates of 6.55 (95% CI, 6.48-6.61) deaths per 100 person-years in men and 5.42 (95% CI, 5.39-5.46) deaths per 100 person-years in women. We observed an association of specific multimorbidity clusters and individual fracture sites with mortality risk. Among individuals in the low-multimorbidity cluster, those with proximal or lower leg fractures experienced excess mortality at 1 year after fracture, with the highest excess mortality found in patients with hip fracture (19.89% [95% CI, 19.06%-20.75%] in men and 11.17% [95% CI, 10.68%-11.67%] in women) (Table 2, Figure 2, and Figure 3). The excess mortality associated with these proximal and lower leg fractures was independent of sex, aging, multimorbidity, and time-related changes in mortality in the comparable population. In contrast, individuals in the low-multimorbidity cluster with forearm, knee, ankle, foot, or hand fractures who were unlikely to have comorbidities at the time of fracture had better survival 1 year after fracture than the age-, sex-, and calendar period–matched general population.

**Table 2.** Excess Mortality 1 Year After Individual Fracture in Selected Sites by Multimorbidity Clusters

Multimorbidity clusters by site	Excess mortality, % (95% CI)	
	Men	Women
Any fracture		
Low-multimorbidity	5.73 (5.48 to 5.99)	2.30 (2.17 to 2.43)
Cardiovascular	18.39 (17.79 to 19.00)	11.35 (11.00 to 11.70)
Diabetic	12.70 (11.69 to 13.76)	8.87 (8.22 to 9.55)
Malignant	26.15 (24.83 to 27.51)	15.81 (15.06 to 16.59)
Hepatic and/or inflammatory	14.36 (13.27 to 15.51)	NA
Hip		
Low-multimorbidity	19.89 (19.06 to 20.75)	11.17 (10.68 to 11.67)
Cardiovascular	29.79 (28.71 to 30.89)	19.90 (19.22 to 20.60)
Diabetic	23.17 (20.96 to 25.52)	17.14 (15.64 to 18.72)
Malignant	40.81 (38.13 to 43.57)	29.29 (27.47 to 31.18)
Hepatic and/or inflammatory	26.50 (23.90 to 29.27)	NA
Femur		
Low-multimorbidity	11.64 (9.36 to 14.23)	10.02 (8.39 to 11.80)
Cardiovascular	26.38 (22.15 to 31.03)	19.04 (16.29 to 22.04)
Diabetic	27.51 (20.02 to 36.65)	18.44 (13.57 to 24.35)
Malignant	51.01 (43.15 to 59.27)	42.02 (36.01 to 48.50)
Hepatic and/or inflammatory	15.47 (9.67 to 23.28)	NA
Pelvis		
Low-multimorbidity	11.68 (8.79 to 15.03)	7.81 (6.44 to 9.30)
Cardiovascular	25.91 (21.28 to 31.05)	15.58 (13.51 to 17.81)
Diabetic	17.48 (9.30 to 29.30)	15.69 (10.98 to 21.49)
Malignant	42.69 (31.19 to 55.88)	23.49 (18.28 to 29.60)
Hepatic and/or inflammatory	14.23 (6.74 to 25.36)	NA
Vertebrae		
Low-multimorbidity	7.32 (6.31 to 8.42)	4.97 (4.20 to 5.82)
Cardiovascular	19.04 (17.02 to 21.19)	11.73 (10.36 to 13.20)
Diabetic	16.61 (12.62 to 21.37)	12.64 (9.65 to 16.18)
Malignant	33.44 (29.13 to 38.11)	25.82 (22.33 to 29.66)
Hepatic and/or inflammatory	20.46 (16.77 to 24.67)	NA
Rib		
Low-multimorbidity	3.08 (2.38 to 3.86)	3.21 (2.11 to 4.50)
Cardiovascular	11.83 (9.94 to 13.93)	10.50 (8.15 to 13.17)
Diabetic	7.37 (4.57 to 11.04)	9.70 (5.35 to 15.73)
Malignant	9.79 (6.33 to 14.35)	15.06 (9.63 to 22.41)
Hepatic and/or inflammatory	10.96 (7.61 to 15.23)	NA
Clavicle		
Low-multimorbidity	1.27 (0.66 to 1.98)	1.47 (0.73 to 2.33)
Cardiovascular	11.23 (8.77 to 14.03)	8.90 (6.78 to 11.33)
Diabetic	6.52 (2.81 to 12.20)	5.42 (1.86 to 10.96)
Malignant	26.47 (20.80 to 33.12)	18.60 (14.06 to 24.16)
Hepatic and/or inflammatory	14.92 (10.75 to 20.17)	NA
Humerus		
Low-multimorbidity	5.12 (4.35 to 5.94)	1.00 (0.69 to 1.33)
Cardiovascular	15.18 (13.48 to 16.99)	7.19 (6.34 to 8.09)
Diabetic	11.95 (9.36 to 14.98)	6.01 (4.70 to 7.52)
Malignant	36.36 (32.47 to 40.51)	14.72 (12.85 to 16.77)
Hepatic and/or inflammatory	10.41 (7.95 to 13.33)	NA
Forearm		
Low-multimorbidity	–0.24 (–0.56 to 0.11)	–1.12 (–1.24 to –1.00)
Cardiovascular	5.44 (4.22 to 6.78)	2.53 (2.02 to 3.07)
Diabetic	3.21 (1.52 to 5.42)	2.13 (1.25 to 3.16)
Malignant	7.59 (5.43 to 10.21)	5.88 (4.89 to 6.97)
Hepatic and/or inflammatory	5.89 (3.90 to 8.40)	NA
Knee		
Low-multimorbidity	–1.21 (–2.05 to 0.4)	–0.85 (–1.38 to –0.13)
Cardiovascular	4.12 (0.82 to 8.81)	2.02 (0.03 to 4.90)
Diabetic	7.37 (2.13 to 16.68)	2.17 (0.10 to 5.99)
Malignant	8.92 (3.13 to 19.49)	6.88 (2.65 to 13.90)
Hepatic and/or inflammatory	2.55 (0.30 to 13.19)	NA
Lower leg		
Low-multimorbidity	1.28 (0.74 to 1.90)	0.87 (0.49 to 1.30)
Cardiovascular	6.17 (4.30 to 8.38)	6.94 (5.57 to 8.46)
Diabetic	4.15 (2.06 to 7.08)	5.76 (3.82 to 8.20)
Malignant	8.72 (5.42 to 13.15)	5.51 (3.43 to 8.29)
Hepatic and/or inflammatory	5.90 (3.22 to 9.71)	NA
Ankle		
Low-multimorbidity	–0.82 (–1.62 to 0.49)	–0.35 (–0.99 to 0.57)
Cardiovascular	3.02 (0.59 to 6.73)	8.19 (4.84 to 12.51)
Diabetic	2.51 (0.33 to 10.76)	4.00 (0.50 to 10.19)
Malignant	3.55 (0.43 to 15.47)	5.59 (1.41 to 13.81)
Hepatic and/or inflammatory	5.00 (0.24 to 21.90)	NA
Hand		
Low-multimorbidity	–0.15 (–0.53 to 0.30)	–1.12 (–1.33 to –0.87)
Cardiovascular	2.76 (1.36 to 4.43)	2.04 (0.96 to 3.31)
Diabetic	4.44 (1.78 to 8.30)	2.63 (0.92 to 5.01)
Malignant	6.50 (3.81 to 10.12)	3.13 (1.47 to 5.43)
Hepatic and/or inflammatory	4.37 (1.88 to 7.95)	NA
Foot		
Low-multimorbidity	–0.70 (–1.03 to –0.27)	–0.65 (–0.89 to –0.37)
Cardiovascular	2.02 (0.41 to 4.17)	2.45 (1.29 to 3.86)
Diabetic	1.78 (1.01 to 4.52)	1.73 (0.30 to 3.93)
Malignant	4.29 (1.33 to 9.17)	5.15 (3.12 to 7.92)
Hepatic and/or inflammatory	3.48 (0.93 to 7.91)	NA

Abbreviation: NA, not applicable.

In those with distal fractures without excess fracture-related mortality, the presence of multimorbidity itself was associated with increased mortality risk, suggesting that multimorbidity contributes to mortality risk independently of sex, aging, and fracture. For instance, men with a forearm fracture in the diabetic cluster had 1-year excess mortality of 3.21% (95% CI, 1.52%-5.42%); in the cardiovascular cluster, 5.44% (95% CI, 4.22%-6.78%); in the hepatic and/or inflammatory cluster, 5.89% (95% CI, 3.90%-8.40%); and in the malignant cluster, 7.59% (95% CI, 5.43%-10.21%).

Multimorbidity in patients with proximal fractures resulted in a compounding association with mortality, much greater than either fracture or comorbidity alone. The most profound compounding association was found for patients in the malignant cluster, with excess mortality ranging from 36.36% (95% CI, 32.47%-40.51%) to 51.01% (95% CI, 43.15%-59.27%) in men and 14.72% (95% CI, 12.85%-16.77%) to 42.02% (95% CI, 36.01%-48.50%) in women with hip, femur, pelvis, or humerus fracture. For example, the 1-year excess mortality for men with hip fracture in the malignant cluster was 40.81% (95% CI, 38.13%-43.57%), substantially greater than hip fracture mortality among men in the low-multimorbidity cluster (19.89% [95% CI, 19.06%-20.75%]) or among men in the malignant cluster with forearm (7.59% [95% CI, 5.43%-10.21%]) or hand (6.50% [95% CI, 3.81%-10.12%]) fractures. This pattern was also observed for other multimorbidity clusters and other proximal and lower leg fractures (Table 2 and Figures 2 and 3).

## Discussion

Both multimorbidity and fractures are becoming increasingly prevalent as the population ages. This cohort study identified distinct multimorbidity clusters in patients with fractures and quantified the interaction between multimorbidity and specific fracture sites associated with postfracture excess mortality, which to our knowledge has not yet been investigated. The association of multimorbidity with mortality risk was substantial and specific to both fracture site and multimorbidity cluster. Importantly, the combination of specific multimorbidity clusters and fractures already associated with excess mortality resulted in a compounding of mortality risk. Beyond their specific relevance to fractures, these findings shed light on the more general problem of multimorbidity that appears to compound the impact of the specific condition being investigated, in this case fracture at different sites.

We found comorbidities were grouped into distinct, clinically meaningful clusters that captured most of the advanced eponymous disease in each. The diabetic cluster illustrates this point. Although only a minority of patients with diabetes have documented diabetes complications, nearly all of those who died were in the diabetic cluster. The diabetic cluster therefore represents not those who simply meet the diagnostic criteria for diabetes, but rather those who have evidence of advanced or difficult-to-control disease, as manifested by concomitant microvascular or macrovascular disorders. It is also worth noting that coexisting renal disease was distributed between the cardiovascular and diabetic clusters. Similarly, the malignant cluster and hepatic and/or inflammatory cluster, each of which constituted only 5% of the population, each captured almost all metastatic cancers and all hepatic disorders and rheumatoid arthritis cases, respectively, indicating that the multimorbidity clusters were robust and unique. These clusters represent additional stratification beyond simple additive summation (eg, using the Charlson Comorbidity Index).^14^ Two individuals with the same number but different combinations of diseases might have different risk profiles and pathophysiological mechanisms, thus requiring different interventions. Unsurprisingly, studies using additive summation to define multimorbidity have failed to document the favorable impact of proposed interventions for patients with multimorbidity.^22,23^

Our study extends and generalizes previous findings^10^ that history of heart failure, chronic obstructive pulmonary disease, dementia, or malignant disease is associated with added mortality risk after hip fracture. In our study, specific clusters of comorbidities were associated with excess postfracture mortality, more so than the risk due to fracture and aging. Most importantly, specific combinations of multimorbidity and fracture sites displayed a compound mortality risk increase, greater than that attributable to multimorbidity or fracture alone. For instance, the excess mortality of patients in the malignant cluster with hip fracture was approximately 2- and 6-fold greater than that of either hip fracture or belonging to the malignant cluster, respectively. The compounding mortality risk resulting from the combination between multimorbidity and fracture highlights the importance of preventing fracture in these high-risk patients.

Using fracture as an example, our findings shed light on the more general problem of multimorbidity and show that more thorough assessment of comorbidity yields important insights. Our statistical strategy may be applicable to many disease settings in which sentinel events occur in the setting of preexisting chronic diseases. Identifying multimorbidity clusters offers the prospect of focusing future guideline development, as well as natural history and mechanistic studies of the interaction of multimorbidity clusters with specific conditions. These insights have the potential to guide a shift from a single-disease approach to individualized management informed by specific multimorbidity profiles.^23^ It has already been noted that the coexistence of chronic health disorders may make the very high-risk nature of heart failure with reduced ejection fraction much more apparent in clinical practice, possibly resulting in better prioritization of preventive efforts.^24^ Identification of the specific multimorbidity cluster also provides additional information about other coexisting diseases, assisting physicians to account for the patient’s health goals, thereby aligning care options with those goals.^25^ For instance, the health outcome goals for a patient in the diabetic cluster should be formulated to account for not only diabetes, but also cardiovascular and chronic kidney diseases that commonly coexist in the cluster. Furthermore, differences between the patients in the low-multimorbidity cluster with diabetes and patients in the diabetic cluster also help in understanding conflicting data regarding the association of diabetes with fractures. Our findings suggest that although patients with uncomplicated diabetes and good control may experience postfracture mortality risk similar to that of the general population, those with advanced disease may be uniquely vulnerable after fracture and so benefit from a more aggressive approach to fracture prevention. This holistic approach has been shown to be superior in management of patients with multimorbidity^23^ to considering coexisting diseases separately, particularly when guideline recommendations conflict.^26^

### Strengths and Limitations

Our findings feature a nationwide study population of patients with fractures and robust diagnostic data, minimizing potential selection bias and misclassification^27^ and allowing us to examine mortality risk after fracture at various sites. Latent class analysis has been shown to be superior to count-based methods of measuring multimorbidity^28^ and to alternative clustering methods.^16,17^ It provides rigorous statistical tests to assess model fit and class separation and specifies formal criteria to optimize cluster construction.^17^ Relative survival analysis allows the joint and separate contributions of both the specific multimorbidity cluster and specific fracture site to mortality to be distinguished from potential confounders.^20^

Our study has several limitations. Latent class analysis by itself does not account for disease severity. However, comorbidities with different severity are classified separately in our study.^12^ Registry-based data are prone to variable accuracy and omission of nonmedical factors.^29^ Nevertheless, all essential study variables were systematically obtained from the Danish NHDR, which includes excellent, complete medical records and precise diagnoses for all individuals living in Denmark since 1995.^30,31^ The Danish NHDR might be unable to capture mild fractures or comorbidities not requiring medical attention. Given the high concordance between self-reported fractures and those registered in the NHDR^32^ and high positive predictive value of the self-reported comorbidities,^33^ it is unlikely that the mild fractures and comorbidities unregistered in the NHDR would substantially modify the findings.

## Conclusions

The findings of this cohort study suggest that concomitant illnesses naturally clustered into distinct multimorbidity clusters were associated with excess mortality after fracture. The combination of multimorbidity and a proximal fracture resulted in a compound increase in mortality, strongly indicating the need for more comprehensive approaches for high-risk patients. Our findings further support the need for future studies that address postfracture care of high-risk patients and examine the association between multimorbidity clusters and other sentinel health events.

## Notes

Supplement. **eMethods.** Latent Class Analysis and Relative Survival Analysis
eReferences
**eTable 1.** List of *ICD-10* Codes Used to Define Specific Fractures and Comorbidities of Interest **eTable 2.** Number of Comorbidities and Deaths by Specific Fracture Sites **eTable 3.** The Parameters of Latent Class Models **eTable 4.** Probabilistic Distribution of All Comorbidities of Interest Within Each Multimorbidity Cluster **eFigure 1.** Flowchart of Follow-up **eFigure 2.** The Model’s Fit Indices of the Latent Class Models
