# Supplementary material for: Association of Multimorbidity and Excess Mortality After Fractures Among Danish Adults
Source: JAMA Netw Open. 2022 Oct 10;5(10):e2235856. doi: 10.1001/jamanetworkopen.2022.35856 (PMC9552889; doi:10.1001/jamanetworkopen.2022.35856)
Supplement: Supplement. — eMethods. Latent Class Analysis and Relative Survival Analysis eReferences eTable 1. List of ICD-10 Codes Used to Define Specific Fractures and Comorbidities of Interest eTable 2. Number of Comorbidities and Deaths by Specific Fracture Sites eTable 3. The Parameters of Latent Class Models eTable 4. Probabilistic Distribution of All Comorbidities of Interest Within Each Multimorbidity Cluster eFigure 1. Flowchart of Follow-up eFigure 2. The Model’s Fit Indices of the Latent Class Models [file jamanetwopen-e2235856-s001.pdf]

Supplementary Online Content

Tran T, Bliuc D, Ho-Le T, et al. Association of multimorbidity and excess mortality after fractures among Danish adults. *JAMA Netw Open*. 2022;5(10):e2235856. doi:10.1001/jamanetworkopen.2022.35856

eMethods. Latent Class Analysis and Relative Survival Analysis
eReferences
eTable 1. List of *ICD-10* Codes Used to Define Specific Fractures and Comorbidities of Interest
eTable 2. Number of Comorbidities and Deaths by Specific Fracture Sites
eTable 3. The Parameters of Latent Class Models
eTable 4. Probabilistic Distribution of All Comorbidities of Interest Within Each Multimorbidity Cluster
eFigure 1. Flowchart of Follow-up
eFigure 2. The Model's Fit Indices of the Latent Class Models

This supplementary material has been provided by the authors to give readers additional information about their work.

© 2022 Tran T et al. *JAMA Network Open*.

eMethods. Latent Class Analysis and Relative Survival Analysis

Latent class analysis

A latent class analysis (LCA)[1] is a cluster analysis that groups binary objects so that objects in the same group are more similar to each other than those in other groups using their estimated probabilities of belonging to a specific group. This statistical clustering technique utilises maximum likelihood estimation to assimilate the observed specific comorbidities into unobserved classes that are statistically distinct and clinically meaningful[2]. Importantly, LCA allows the comparison between all possible combinations of the variables of interest, which were the specific health conditions in our analysis to be statistically tested, making the selection of optimal clusters rigorous, objective and reproducible.[2]

Latent class analysis has been shown to be superior to the counts-based methods of measuring multimorbidity, which are usually not possible to distinguish between individuals with the same number but different types of diseases[3]. LCA is also more robust than the conventional clustering techniques[2 4] as not only does LCA provide rigorous statistical tests to assess model fit, class separation, but also formal criteria to make decisions to select the optimal clusters. LCA has been found to be also associated with significantly lower rate of misclassification than the k-means method, one of the most commonly used clustering methods (1.3% vs. 8%, P< 0.01)[5]. However, as LCA assigns the participants to cluster based on their probability of being in the clusters[1 2], a risk of misclassification bias in LCA, though lower than the conventional clustering techniques cannot be completely eliminated. Additionally, the name of the identified clusters, usually assigned by researchers for convenience in interpretation sometimes leads to a "naming fallacy"[6] wherein the cluster name might not accurately or fully reflect the cluster nature due to complexity of the cluster.

We conducted LCA[1] to determine the unique clusters of comorbidities presented at the time of fracture. Technically, we first developed a series of models in which we varied the number of latent classes from 2 to 10. We optimized the number of latent classes to provide the best fit for the data and the best separation among classes. Latent class enumeration was conducted without covariates to prevent problems related to class overestimation[7]. The optimal number of classes was chosen based on three criteria: (i) data fit, (ii) separation capacity and (iii) smallest meaningful class. We used the statistical model fit indices including log likelihood plot where the log likelihood starts to level off and other criteria, such as adjusted Bayesian Information Criterion (BIC) and Akaike Information Criterion

© 2022 Tran T et al. *JAMA Network Open*.

(AIC) in which the smaller value indicates better fit[8]. An entropy statistic was used to quantify the degree of separation between latent classes, with the level of ≥ 0.6 suggesting good between-group separation[9], whereas the smallest meaningful estimated class proportion was expected to be at least 5%[8]. Once the optimal latent class model was selected, each participant was assigned to the "best-fit" cluster for which he or she had the highest computed probability of membership.

Relative survival analysis

Relative survival analysis was originally developed to determine excess mortality attributable to a specific cancer by comparing the observed all-cause mortality rate in the cancer cohort with the expected mortality rate in a comparative age-, sex- and calendar period-matched general population. All-cause deaths in the cancer cohort result from two sources: the cancer *per se* and other causes; whereas the expected background time-related mortality rate in the comparative general cohort is assumed to reflect the contribution of "other causes" to mortality over time. As a result, the excess mortality is then considered a robust measure of mortality attributable to the cancer or a disease of interest[10]. Cause-specific mortality data are not needed in a relative survival analysis, making it especially relevant for examining the contribution of fracture to mortality as a fracture is rarely recognized as a contributing cause of death[11 12].

We performed relative survival analysis to quantify excess mortality attributable to the interaction between specific multimorbidity clusters and individual fracture sites under similar assumptions originally used in oncology research[10]. Excess mortality for patients in a specific multimorbidity cluster who fractured at a specific site, calculated as 1 minus its relative survival ratio can be interpreted as the proportion of patients who would die due to the combination between the specific comorbidity cluster and the individual fracture site. The relative survival ratio is the ratio of observed survival among the cohort of individuals in a specific multimorbidity cluster who fractured at a specific site to the expected survival in a comparative age-, sex- and calendar period-matched general population[10]. The observed survival was calculated using all-cause deaths of the fracture cohort; whereas the expected survival is the survival probability of similar individuals from the general population of the same age, sex and calendar period as the fracture cohort[10]. We used the Ederer II method[13] to estimate the expected mortality rate from the Danish population life tables stratified by sex, age and calendar period from the Human Mortality Database[14]. An excess mortality of zero

© 2022 Tran T et al. *JAMA Network Open*.

indicates that the mortality rate observed in the cohort of patients with specific fracture in a specific cluster of comorbidities is not different from that in a comparative background population and hence that no excess mortality is attributable to the combination of the cluster of comorbidity and the fracture site.

eReferences

1. Linzer DAL, J. B. poLCA: An R Package for Polytomous Variable Latent Class Analysis. *J Stat Soft* 2011;42(10)

2. Hagenaars JAM, A. L. Applied latent class analysis: Cambridge University Press 2002.

3. Harrison C, Britt H, Miller G, et al. Examining different measures of multimorbidity, using a large prospective cross-sectional study in Australian general practice. *BMJ open* 2014;4(7):e004694.

4. Busija L, Lim K, Szoeke C, et al. Do replicable profiles of multimorbidity exist? Systematic review and synthesis. *Eur J Epidemiol* 2019;34(11):1025-53.

5. Magidson J, Vermunt J. Latent class models for clustering: a comparison with K-means. *Can J Mark Res* 2002;20:37-44.

6. Weller BE, Bowen NL, Faubert, SL. Latent Class Analysis: A Guide to Best Practice. *J Black Psychol* 2020;46(4):287-311.

7. Vermunt JK. Latent class modeling with covariates: two improved three-step approaches. *Polit Anal* 2010;18(4):450-69.

8. Nylund KLA, T.; Muthen, B.O. Deciding on the number of classes in latent class analysis and growth mixture modeling: a Monte Carlo simulation study. *Struct Equ Modelling* 2007;14:535-69.

9. Asparouhov TM, B. Auxiliary variables in mixture modeling: Three-step approaches using Mplus. *Struct Equ Modelling* 2014;21(3):329-41.

10. Dickman PW, Sloggett A, Hills M, et al. Regression models for relative survival. *Stat Med* 2004;23(1):51-64.

11. Calder SJ, Anderson GH, Gregg PJ. Certification of cause of death in patients dying soon after proximal femoral fracture. *BMJ* 1996;312(7045):1515.

12. Hindmarsh DM, Hayen A, Finch CF, et al. Relative survival after hospitalisation for hip fracture in older people in New South Wales, Australia. *Osteoporos Int* 2009;20(2):221-9.

13. Ederer F, Axtell LM, Cutler SJ. The relative survival rate: a statistical methodology. *J Natl Cancer Inst Monogr* 1961;6:101-21.

14. Human Mortality Database. University of California, Berkeley (USA), and Max Planck Institute for Demographic Research (Germany). Available at: www.mortality.org.

© 2022 Tran T et al. *JAMA Network Open*.

eTable 1. List of *ICD-10* Codes Used to Define Specific Fractures and Comorbidities of Interest

**Conditions**	***ICD-10* codes**
**Fractures:**	
Proximal fractures:	
Hip	S72.0- S72.2
Femur	S72.3-S72.9
Pelvis	S32.3-S32.5
Vertebrae	S22.0, S22.1, S32.0, S32.2, S32.7, S32.8, T08.x
Humerus	S42.x
Rib	S22.3-S22.4
Clavicle	S42.0
Distal fractures:	
Forearm	S52.x
Lower leg	S82.2-S82.8
Knee	S82.0
Ankle	S82.5-S82.6
Foot	S92.0-S92.3, S92.7, S92.9
Hand	S62.0-S62.4, S62.8
**Comorbidities:**	
Myocardial infarct	I21.x, I22.x, I25.2
Congestive heart failure	I09.9, I11.0, I13.0, I13.2, I25.5, I42.0, I42.5 - I42.9, I43.x, I50.x, P29.0
Peripheral vascular disease	I70.x, I71.x, I73.1, I73.8, I73.9, I77.1, I79.0, I79.2, K55.1, K55.8, K55.9, Z95.8, Z95.9
Cerebrovascular disease	G45.x, G46.x, H34.0, I60.x - I69.x
Cardiac valvular disease	A52.0, I05.x - I08.x, I09.1, I09.8, I34.x - I39.x, Q23.0 - Q23.3, Z95.2 - Z95.4
Cardiac arrhythmias	I44.1 - I44.3, I45.6, I45.9, I47.x - I49.x, R00.0, R00.1, R00.8, T82.1, Z45.0, Z95.0
Diabetes without chronic complication	E10.0, E10.1, E10.6, E10.8, E10.9, E11.0, E11.1, E11.6, E11.8, E11.9, E12.0, E12.1, E12.6, E12.8, E12.9, E13.0, E13.1, E13.6, E13.8, E13.9, E14.0, E14.1, E14.6, E14.8, E14.9

© 2022 Tran T et al. *JAMA Network Open*.

**Conditions**	***ICD-10* codes**
Diabetes with chronic complication	E10.2 - E10.5, E10.7, E11.2 - E11.5, E11.7, E12.2 - E12.5, E12.7, E13.2 - E13.5, E13.7, E14.2 - E14.5, E14.7
Any malignancy, including lymphoma and leukaemia, except malignant neoplasm of skin	C00.x - C26.x, C30.x - C34.x, C37.x - C41.x, C43.x, C45.x - C58.x, C60.x - C76.x, C81.x - C85.x, C88.x, C90.x - C97.x
Metastatic solid tumour	C77.x - C80.x
Rheumatic/Rheumatoid arthritis or collagen vascular disease	L94.0, L94.1, L94.3, M05.x, M06.x, M31.5, M32.x - M34.x, M08.x, M12.0, M12.3, M30.x, M31.0 - M31.3, M32.x - M35.x, M36.0, M45.x, M46.1, M46.8, M46.9
Mild liver disease	B18.x, K70.0 - K70.3, K70.9, K71.3 - K71.5, K71.7, K73.x, K74.x, K76.0, K76.2 - K76.4, K76.8, K76.9, Z94.4
Moderate or severe liver disease	I85.0, I85.9, I86.4, I98.2, K70.4, K71.1, K72.1, K72.9, K76.5, K76.6, K76.7
Hypertension	I10.x, I11.x - I13.x, I15.x
Chronic pulmonary disease	I27.8, I27.9, J40.x - J47.x, J60.x - J67.x, J68.4, J70.1, J70.3
Pulmonary circulation disorders	I26.x, I27.x, I28.0, I28.8, I28.9
Dementia	F00.x - F03.x, F05.1, G30.x, G31.1
Psychoses	F20.x, F22.x - F25.x, F28.x, F29.x, F30.2, F31.2, F31.5
Depression	F20.4, F31.3 - F31.5, F32.x, F33.x, F34.1, F41.2, F43.2
Neurological disorders	G10.x - G13.x, G20.x - G22.x, G25.4, G25.5, G31.2, G31.8, G31.9, G32.x, G35.x - G37.x, G40.x, G41.x, G93.1, G93.4, R47.0, R56.x
Hemiplegia or paraplegia	G04.1, G11.4, G80.1, G80.2, G81.x, G82.x, G83.0 - G83.4, G83.9
Peptic ulcer disease	K25.x - K28.x
Chronic kidney disease	I12.0, I13.1, N03.2 - N03.7, N05.2 - N05.7, N18.x, N19.x, N25.0, Z49.0 - Z49.2, Z94.0, Z99.2
Hypothyroidism	E00.x - E03.x, E89.0
Coagulopathy	D65 - D68.x, D69.1, D69.3 - D69.6
Obesity	E66.x
Unintended weight loss	E40.x - E46.x, R63.4, R64
Fluid and electrolyte disorders	E22.2, E86.x, E87.x
Anaemia	D50.0, D50.8, D50.9, D51.x - D53.x

© 2022 Tran T et al. *JAMA Network Open*.

**Conditions**	***ICD-10* codes**
Alcohol abuse	F10, E52, G62.1, I42.6, K29.2, K70.0, K70.3, K70.9, T51.x, Z50.2, Z71.4, Z72.1
Drug abuse	F11.x - F16.x, F18.x, F19.x, Z71.5, Z72.2
AIDS/HIV	B20.x - B22.x, B24.x

© 2022 Tran T et al. *JAMA Network Open*.

eTable 2. Number of Comorbidities and Deaths by Specific Fracture Sites

	Men						Women					
	**Number of fractures**	**Age at fracture (years): Mean (SD)**	**Number of co-morbidities: Median (IQR)**	**Number of deaths**	**Follow-up time (person-years)**	**Incidence of death per 100 person-years (95% CI)**	**Number of fractures**	**Age at fracture (years): Mean (SD)**	**Number of co-morbidities: Median (IQR)**	**Number of deaths**	**Follow-up time (person-years)**	**Incidence of death per 100 person-years (95% CI)**
Hip	25693	79.5 (9.7)	2 (1, 4)	16873	107767	15.0 (14.8, 15.2)	51666	82.1 (9.2)	2 (1, 3)	31806	259059	11.4 (11.3, 11.5)
Femur	1920	74.5 (10.8)	2 (1, 4)	1006	10467	9.6 (9.0, 10.2)	3530	78.9 (10.9)	1 (0, 3)	1924	19320	9.9 (9.5, 10.3)
Pelvis	1303	77.1 (10.9)	2 (1, 4)	752	6463	11.6 (10.8, 12.5)	4924	81.2 (10.0)	2 (1, 3)	2894	26759.5	10.7 (10.3, 11.1)
Vertebra	6932	72.7 (10.5)	2 (1, 3)	3136	41737	7.5 (7.2, 7.7)	9753	76.6 (10.6)	2 (0, 3)	4669	60291	7.7 (7.4, 7.9)
Rib	6879	69.7 (10.4)	1 (0, 3)	2368	50177	4.7 (4.5, 4.9)	3163	74.0 (11.9)	1 (0, 3)	1221	22284	5.5 (5.2, 5.8)
Clavicle	5197	68.1 (10.5)	1 (0, 2)	1629	39723	4.1 (3.9, 4.3)	4464	72.7 (11.5)	1 (0, 2)	1584	31754	5.0 (4.7, 5.2)
Humerus	10117	72.6 (10.7)	2 (1, 3)	4822	62339	7.7 (7.4, 7.9)	28298	74.4 (10.5)	1 (0, 2)	10327	202649	5.0 (4.9, 5.1)
Forearm	15255	69.4 (10.2)	1 (0, 2)	4663	120702	3.8 (3.7, 3.9)	68310	72.0 (10.3)	1 (0, 2)	18457	559370	3.1 (3.1, 3.2)
Knee	1387	70.2 (10.1)	1 (0, 3)	448	10886	4.1 (3.7, 4.5)	2609	71.3 (9.7)	1 (0, 2)	629	21084.5	3.0 (2.8, 3.2)
Lower leg	6302	67.0 (9.5)	1 (0, 3)	1803	52909	3.4 (3.2, 3.6)	11468	70.1 (10.8)	1 (0, 2)	3139	94303	3.3 (3.2, 3.4)
Ankle	1081	67.6 (9.8)	1 (0, 2)	272	9080	3.0 (2.6, 3.7)	1936	70.0 (10.6)	1 (0, 2)	496	15726	3.2 (2.0, 3.4)
Hand	8309	67.7 (10.1)	1 (0, 2)	2176	70258	3.1 (3.0, 3.2)	12471	69.6 (10.2)	1 (0, 2)	2681	108102.5	2.5 (2.4, 2.6)
Foot	4997	65.3 (8.7)	1 (0, 2)	1069	44132.5	2.4 (2.3, 2.6)	9907	67.8 (9.6)	1 (0, 2)	1900	86131	2.2 (2.1, 2.3)

© 2022 Tran T et al. *JAMA Network Open*.

eTable 3. The Parameters of Latent Class Models

	2-class	3-class	4-class	5-class	6-class	7-class	8-class	9-class	10-class
**Men (n=95372)**									
Number of parameters	65	98	131	164	197	230	263	296	329
Degree of freedom	95307	95274	95241	95208	95175	95142	95109	95076	95043
Maximal Log Likelihood	-595242	-588659	-583536	-578908	-576199	-575045	-574213	-573668	-573318
AIC	1190613	1177514	1167333	1158144	1152792	1150551	1148952	1147927	1147294
BIC	1191228	1178442	1168573	1159696	1154657	1152728	1151442	1150729	1150408
Adjusted BIC	1190514	1177365	1167134	1157894	1152493	1150201	1148552	1147477	1146793
Likelihood ratio	117347.5	104182.9	93936	84680.4	79263	76955.4	75290.6	74199.9	73500.8
Smallest class	31.3%	5.9%	2.0%	5.1%	2.0%	2.0%	2.0%	2.1%	1.2%
Entropy	0.673	0.686	0.711	0.725	0.677	0.677	0.639	0.641	0.637
**Women (n=212498)**									
Number of parameters	65	98	131	164	197	230	263	296	329
Degree of freedom	212433	212400	212367	212334	212301	212268	212235	212202	212169
Maximal Log Likelihood	-1123516	-1114338	-1105428	-1100572	-1096786	-1095184	-1093782	-1092826	-1091965
AIC	2247161	2228871	2211118	2201472	2193966	2190829	2188089	2186244	2184587
BIC	2247829	2229877	2212463	2203156	2195989	2193190	2190789	2189283	2187965
Adjusted BIC	2247065	2228725	2210923	2201228	2193673	2190486	2187698	2185803	2184098
Likelihood ratio	156969.8	138613.7	120794.8	111082.6	103510.6	100307	97501.5	95590.4	93867.8
Smallest class	26.5%	4.4%	5.0%	3.8%	0.9%	0.9%	1.0%	0.9%	0.8%
Entropy	0.643	0.651	0.697	0.653	0.645	0.626	0.600	0.590	0.591

AIC= Akaike Information Criterion; BIC= Bayesian Information Criterion

© 2022 Tran T et al. *JAMA Network Open*.

eTable 4. Probabilistic Distribution of All Comorbidities of Interest Within Each Multimorbidity Cluster

	Men					Women			
**Comorbidities**	**Low multi-morbidity (60.5%)**	**Cardio-vascular (23.7%)**	**Diabetic (5.6%)**	**Malignant (5.1%)**	**Hepatic/ Inflammatory (5.1%)**	**Low multi-morbidity (66.5%)**	**Cardio-vascular (23.5%)**	**Diabetic (5.0%)**	**Malignant (5.0%)**
Myocardial infarct	3.7%	26.9%	27.0%	7.5%	11.7%	1.6%	16.4%	20.3%	3.7%
Congestive heart failure	2.0%	38.2%	35.4%	8.0%	17.5%	1.6%	28.9%	29.6%	5.6%
Peripheral vascular disease	3.6%	19.9%	33.8%	8.3%	12.3%	2.4%	13.5%	21.4%	5.8%
Cerebrovascular disease	12.1%	40.9%	39.4%	16.4%	25.6%	8.9%	35.4%	33.9%	12.56%
Cardiac valvular disease	0.3%	8.4%	5.7%	1.9%	3.6%	0.2%	8.1%	5.5%	1.2%
Cardiac arrhythmias	2.7%	34.4%	23.1%	9.1%	14.9%	1.9%	25.9%	19.1%	5.9%
Diabetes without chronic complications	4.3%	10.2%	96.5%	9.9%	15.8%	3.0%	7.4%	93.0%	8.0%
Diabetes with chronic complications	0.4%	0.5%	77.1%	0.4%	3.1%	0.2%	0.03%	57.8%	0.2%
Any malignancy, except malignant neoplasm of skin	12.2%	20.0%	21.3%	100.0%	21.7%	10.6%	16.0%	20.5%	99.9%
Metastatic solid tumour	0.4%	0.9%	3.0%	48.9%	3.5%	0.3%	0.8%	2.3%	4.34%
Rheumatic/ Rheumatoid arthritis or collagen vascular disease	0.7%	1.0%	4.0%	0.9%	35.2%	4.2%	9.6%	10.0%	6.4%
Mild liver disease	0.8%	0.7%	4.6%	1.9%	33.3%	0.7%	3.2%	5.1%	2.7%
Moderate/severe liver disease	0.1%	0.1%	1.7%	0.5%	17.7%	0.1%	1.2%	1.8%	0.9%
Hypertension	4.2%	35.4%	46.7%	15.7%	19.5%	4.8%	37.2%	46.6%	16.4%
Chronic pulmonary disease	8.2%	30.2%	23.6%	17.0%	24.5%	7.7%	25.5%	23.1%	18.6%
Pulmonary circulation disease	0.3%	2.7%	2.0%	1.5%	1.4%	0.2%	2.7%	1.9%	1.2%
Dementia	6.1%	17.5%	11.3%	2.9%	9.4%	6.0%	19.3%	12.0%	3.4%
Psychoses	0.3%	0.7%	0.5%	0.2%	7.7%	0.2%	0.8%	0.6%	0.3%
Depression	0.5%	5.9%	4.2%	1.8%	5.5%	0.5%	8.8%	4.4%	2.3%
Neurological disorder	3.3%	8.9%	5.1%	2.7%	6.8%	1.7%	6.0%	3.8%	2.5%

© 2022 Tran T et al. *JAMA Network Open*.

	Men					Women			
**Comorbidities**	**Low multi-morbidity (60.5%)**	**Cardio-vascular (23.7%)**	**Diabetic (5.6%)**	**Malignant (5.1%)**	**Hepatic/ Inflammatory (5.1%)**	**Low multi-morbidity (66.5%)**	**Cardio-vascular (23.5%)**	**Diabetic (5.0%)**	**Malignant (5.0%)**
Hemi/paraplegia	0.6%	1.4%	1.8%	1.6%	1.4%	0.3%	0.8%	0.8%	1.0%
Peptic ulcer disease	3.8%	14.5%	13.7%	8.5%	20.9%	3.2%	14.3%	12.1%	7.0%
Chronic kidney disease	0.9%	13.0%	23.2%	5.1%	7.8%	0.4%	6.6%	1.6%	3.2%
Hypothyroidism	0.2%	1.3%	1.5%	0.6%	0.9%	0.6%	4.8%	6.0%	2.2%
Coagulopathy	0.1%	1.0%	1.0%	0.8%	2.2%	0.1%	0.8%	0.9%	0.4%
Obesity	0.3%	1.9%	9.0%	1.0%	2.1%	0.4%	1.9%	11.1%	1.9%
Unintended weight loss	0.1%	1.3%	1.2%	1.0%	2.0%	0.1%	1.6%	0.8%	0.7%
Fluid/ electrolyte disorder	1.1%	12.2%	9.5%	4.2%	9.0%	1.0%	15.2%	9.5%	4.1%
Anaemia	0.3%	3.8%	3.2%	1.5%	4.4%	0.3%	4.4%	3.2%	1.4%
Alcohol abuse	0.4%	0.5%	2.1%	0.5%	23.6%	0.1%	1.4%	1.3%	0.6%
Drug abuse	0.1%	0.3%	0.3%	0.1%	1.2%	0.03%	0.5%	0.2%	0.1%
HIV/AIDS	0.04%	0.1%	0.1%	0.1%	0.2%	0.0%	0.0%	0.01%	0.01%

Data presented as probabilities (%) of each comorbidity of interest computed from the latent class analysis given the class membership.

© 2022 Tran T et al. *JAMA Network Open*.

eFigure 1. Flowchart of Follow-up

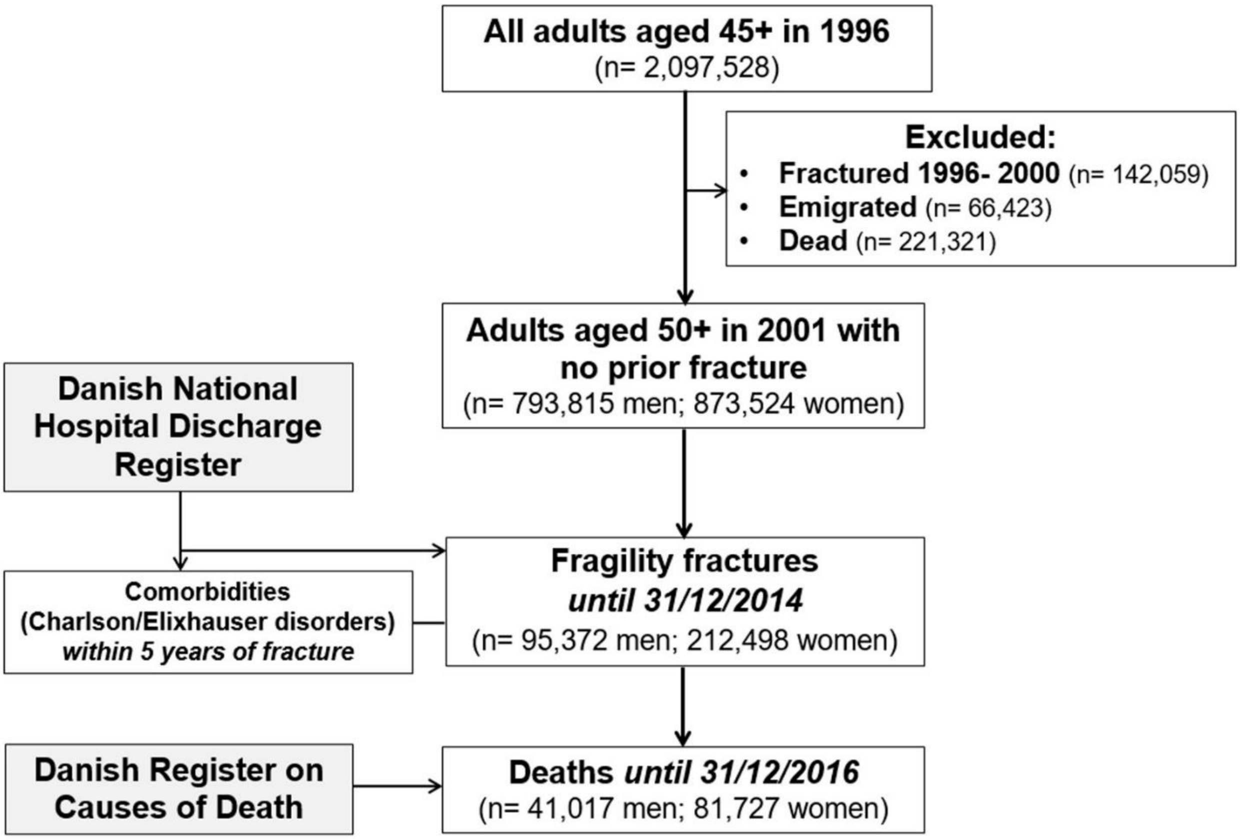

© 2022 Tran T et al. *JAMA Network Open*.

eFigure 2. The Model's Fit Indices of the Latent Class Models

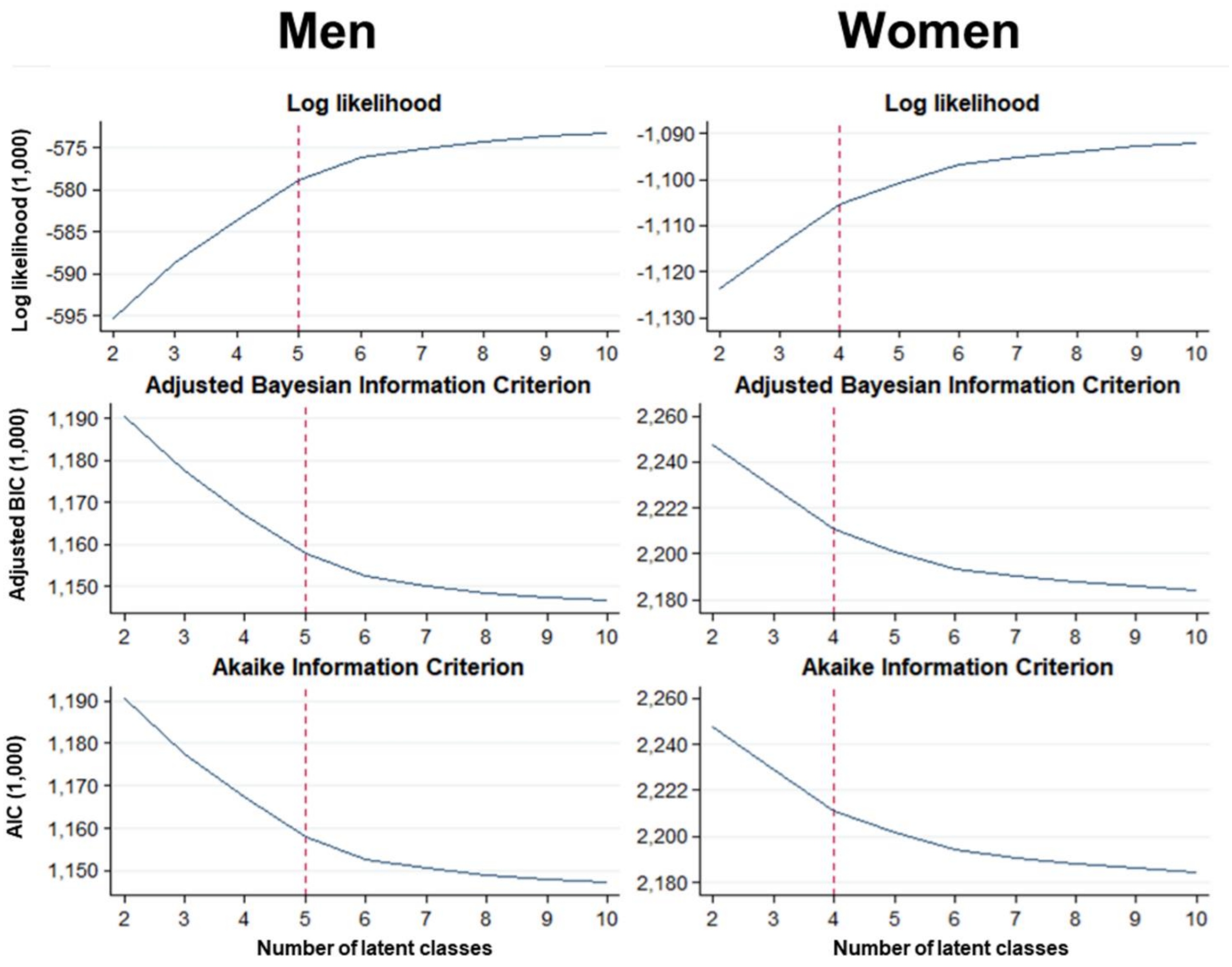

© 2022 Tran T et al. *JAMA Network Open*.
